# Supplementary material for: Early Inhibition of Phosphodiesterase 4B (PDE4B) Instills Cognitive Resilience in APPswe/PS1dE9 Mice
Source: Cells. 2024 Jun 8;13(12):1000. doi: 10.3390/cells13121000 (PMC11201979; doi:10.3390/cells13121000)
Supplement: Supplementary file 1 [file cells-13-01000-s001.zip › cells-2939138-supplementary.pdf]

Supplementary information

Supplementary methods

Bone marrow transplantation and microglial depletion

Recipient 6-weeks-old APPswe/PS1dE9 mice and WT littermates were intraperitoneally anesthetized with ketamine (30 mg/kg) and xylazine (5 mg/kg) diluted in sterile PBS before being lethally γ-irradiated at a dose of 8 gray (Gy). Immediately following irradiation, mice were intravenously injected with 5×10^6 bone marrow cells dissolved in sterile PBS harvested from the tibia and femurs of 6-week-old sex-matched Pde4b$^{+/+}$, Pde4B$^{+/-}$ and Pde4b$^{-/-}$ mice. Mice were given autoclaved tap water supplemented with Neomycin (100 mg/l, Gibco, USA) and Polymyxin B sulfate (60 000 U/l, Sigma-Aldrich, USA) starting two weeks before and ending four weeks after irradiation. One week after bone marrow transplantation (BMT), mice received chow containing the colony-stimulating factor 1 receptor (CSF1R) inhibitor PLX5622 (1200 mg/kg standard chow, Chemgood, USA) for two weeks [1]. The CSF1R is critical for microglial survival, hence its inhibition eliminates the irradiation-damaged, yet still viable, microglia [1]. This combination of BMT and PLX5622 treatment drastically increases the repopulation of the brain with microglia-like cells arising from the donated bone marrow [2].

Supplementary results

Six-week-old mice undergoing bone marrow transplantation have poor survival rates

To further study the effect of microglia bone marrow transplantation of Pde4b$^{+/+}$, Pde4B$^{+/-}$ and Pde4b$^{-/-}$ cells was used to replace the resident microglia in lethally irradiated APPswe/PS1dE9 mice at 6 weeks-of-age. The CSF1R inhibitor PLX5622 was used to block proliferation of the small population of resident microglia that survive irradiation[3]. However, many animals died prematurely (figure S1) and performing behavioral tests and subsequent *post-mortem* analyses were therefore unable to be performed because of the deteriorated health. To investigate why the survival rate of these animals was so poor, a follow-up experiment was conducted. In this experiment, we employed bone marrow transplants and evaluated cardiac health since PDE4B already showed to be involved in cardiac function.

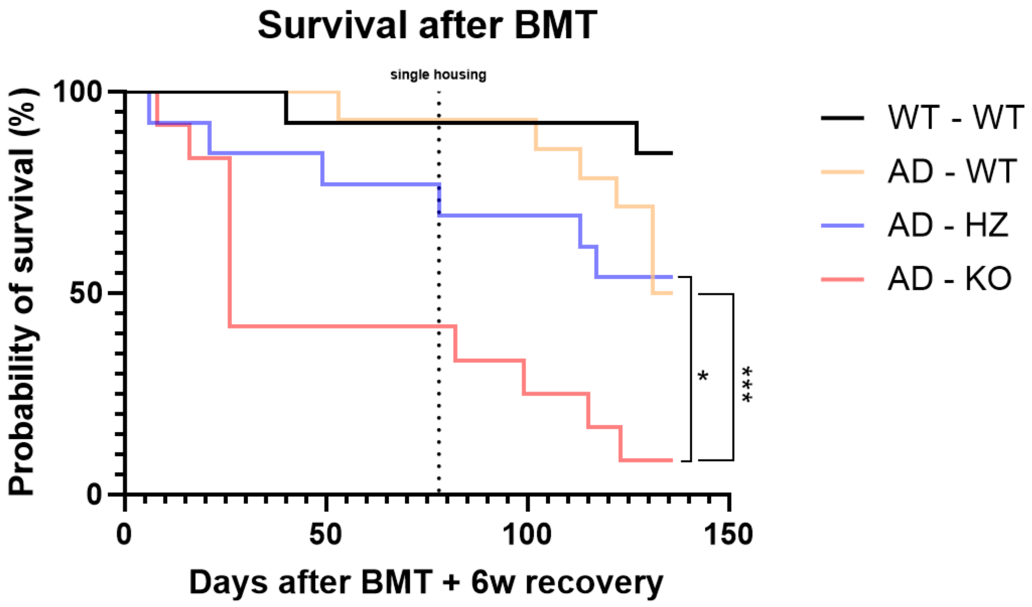

Figure S1: Proportional survival of bone marrow-transplanted (BMT) animals. Survival plot of animals that underwent bone marrow transplantation, divided into groups according to acceptor mouse genotype (WT / APPswe/PS1dE9) and donor mouse Pde4b genotype (WT, HZ, KO). Data are represented as the percentage of surviving animals, starting from the day that the immunodeficient period ended (6 weeks post-procedure). Analyzed by means of Kaplan-Meier survival analysis Mantel-Cox test with Bonferroni correction for multiple comparisons (*$p < 0.05$, ***$p < 0.001$).

Cardiac health deteriorates by bone marrow transplantation, independent of donor genotype

The poor survival rates prompted a follow-up experiment in which cardiac parameters were measured at 4 months-of-age using echocardiography, as described previously, in animals that underwent the BMT procedure [4]. This timepoint was chosen based on the findings of the first bone marrow transplant. At this timepoint, symptoms such as shallow breathing followed by premature death started surfacing. The parameters measured and analyzed to check for congestive heart failure were cardiac output, end diastolic volume, ejection fraction, and wall thickness (figure S2). Cardiac output and end diastolic volume were significantly lowered when comparing transplanted vs. non-transplanted animals (analysis not shown), which could cause pulmonary edema associated with congestive heart failure [5].

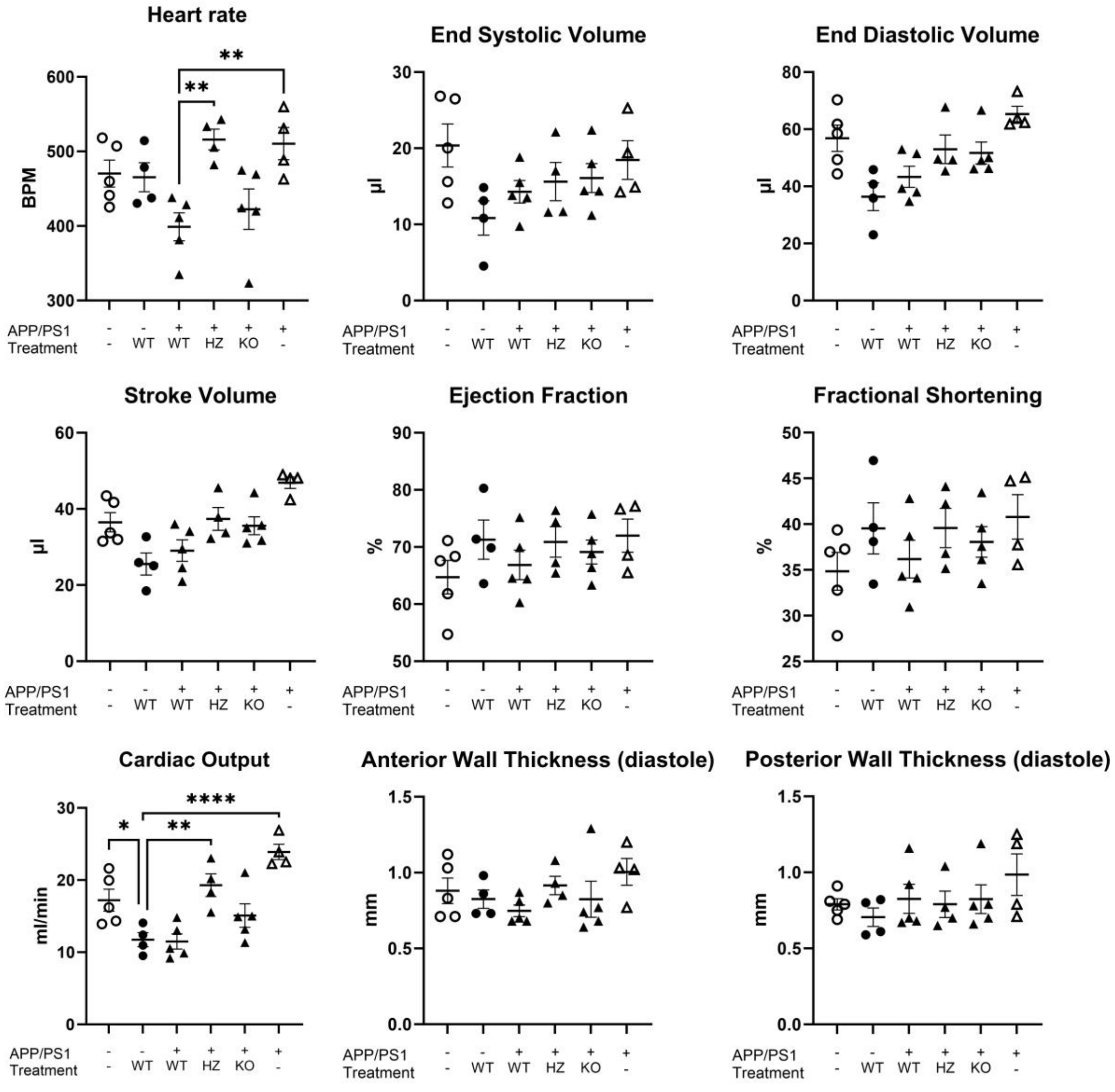

Figure S2: Cardiac parameters measured by means of echocardiography of bone marrow-transplanted (BMT) animals and untreated littermates as controls. Multiple cardiac parameters are plotted per group. Data is represented as mean ± SEM and analyzed by means of one-way ANOVA with Dunnett's multiple comparisons test (*p < 0.05, **p < 0.01, ****p < 0.0001). Treatment represents whether or not animals underwent BMT, and if so, using which donor *Pde4b* genotype. Overall differences between irradiated and non-irradiated animals were analyzed by means of two-way ANOVA with Šídák's multiple comparisons test (data not shown).

To attempt to isolate the microglial effect, bone marrow transplantation of *Pde4b*$^{+/+}$, *Pde4b*$^{+/-}$ and *Pde4b*$^{-/-}$ cells was performed to replace the resident microglia, starting at 6 weeks-of-age. The leakiness of the blood-brain-barrier, caused by the irradiation, allows the newly engrafted circulating bone marrow cells to repopulate in the CNS. When PLX5622 treatment is stopped, the newly introduced microglia-like cells have repopulated the microglial niche to such an extent that they overtake the resident population [3]. In the present study, however, multiple animals have been observed hunching over and having trouble breathing, starting 8 weeks after irradiation which might indicate accumulation of fluid in the thorax, as has been previously described [6]. Working theories on the cause of premature death include the accumulation of circulating immune cells in the lungs or, possibly, congestive heart failure.
These results prompted a follow-up experiment in which cardiac parameters were measured using echocardiography, as described previously, at 4 months of age in animals that underwent the bone marrow transplant procedure [4]. Overall, there were significant differences to be found when comparing irradiated WT and APPswe/PS1dE9 mice to non-irradiated litter mates. There were no significant differences when comparing groups that received bone marrow from different donor groups (*Pde4B*$^{+/+}$, *Pde4B*$^{+/-}$ and *Pde4B*$^{-/-}$) as shown in figure S2. Taken together, the inconclusive results yielded by this experiment warrant further research on the use of bone marrow transplant as a valid model.

References

1. Spangenberg, E.; Severson, P.L.; Hohsfield, L.A.; Crapser, J.; Zhang, J.; Burton, E.A.; Zhang, Y.; Spevak, W.; Lin, J.; Phan, N.Y.; et al. Sustained microglial depletion with CSF1R inhibitor impairs parenchymal plaque development in an Alzheimer's disease model. *Nature Communications* **2019**, *10*, doi:10.1038/s41467-019-11674-z.
2. Xu, Z.; Rao, Y.; Huang, Y.; Zhou, T.; Feng, R.; Xiong, S.; Yuan, T.-F.; Qin, S.; Lu, Y.; Zhou, X.; et al. Efficient Strategies for Microglia Replacement in the Central Nervous System. *Cell Reports* **2020**, *32*, 108041, doi:10.1016/j.celrep.2020.108041.
3. Cronk, J.C.; Filiano, A.J.; Louveau, A.; Marin, I.; Marsh, R.; Ji, E.; Goldman, D.H.; Smirnov, I.; Geraci, N.; Acton, S.; et al. Peripherally derived macrophages can engraft the brain independent of irradiation and maintain an identity distinct from microglia. *The Journal of experimental medicine* **2018**, *215*, 1627-1647, doi:10.1084/jem.20180247.
4. Gao, S.; Ho, D.; Vatner, D.E.; Vatner, S.F. Echocardiography in Mice. *Current Protocols in Mouse Biology* **2011**, *1*, 71-83, doi:10.1002/9780470942390.mo100130.
5. Deluyker, D.; Ferferieva, V.; Driesen, R.B.; Verboven, M.; Lambrichts, I.; Bito, V. Pyridoxamine improves survival and limits cardiac dysfunction after MI. *Scientific reports* **2017**, *7*, doi:10.1038/s41598-017-16255-y.
6. Sharplin, J.; Franko, A.J. A Quantitative Histological Study of Strain-Dependent Differences in the Effects of Irradiation on Mouse Lung during the Intermediate and Late Phases. *Radiation Research* **1989**, *119*, 15, doi:10.2307/3577364.
